# Technology-assisted visceral surgery: a model-based approach for quantifying translation and accessibility across populations and geographies

**DOI:** 10.1007/s11548-026-03723-y

**Published:** 2026-06-06

**Authors:** Sebastian Schmailzl, Jonas Fuchtmann, Alissa Jell, Stephan Schorn, Lukas Bernhard, Lars Wagner, Luca Wegener, Helmut Friess, Dirk Wilhelm, Maximilian Berlet

**Affiliations:** 1 MITI Research Group, TUM School of Medicine and Health, University Hospital Rechts der Isar, Ismaninger Str. 22, 81675 Munich, Bavaria Germany; 2 Department of Surgery, TUM School of Medicine and Health, University Hospital rechts der Isar, Ismaninger Str. 22, 81675 Munich, Bavaria Germany

**Keywords:** Model-guided medicine, Technological translation, Gastrointestinal surgery, Visceral surgery, Surgical robotics, Artificial intelligence, Technology in surgery

## Abstract

**Introduction:**

Technological innovation is rapidly transforming surgical care, yet disparities in access and adoption persist. This study introduces a flexible, population-based model designed to assess and potentially guide the equitable implementation of advanced technologies in visceral (gastrointestinal) surgery, ranging from laparoscopy and basic imaging up to robotic-assisted surgery and artificial intelligence.

**Material and methods:**

We conducted a survey among technologically experienced visceral surgeons to classify current and emerging technologies by perceived complexity and requirement. This classification was used to query a self-developed translation model, which was integrated with demographic, hospital, and geographic data from official German sources. The model quantifies technological maturity and accessibility by calculating travel-to-treat times from the population’s residence to hospitals offering specific technologies. We applied the model to the entire German population and all 16 counties, enabling regional and national comparisons.

**Results:**

Based on the survey results, we constructed a layered classification for grouping surgical technologies with two dimensions, S and D, for surgical procedures and available devices and increasing levels of complexity (1-3). The model identified substantial regional disparities in access to advanced technology-assisted visceral surgery across Germany. While basic technologies (S1–S2, D1–D2) were broadly accessible nationwide, higher-level technologies such as robotic surgery (S3) were concentrated in urban areas. However, some advanced diagnostics (D3) were also available in remote regions. The model effectively quantified these differences from the population’s perspective and demonstrated its potential for objective, population-based assessment of technological translation and accessibility.

**Conclusion:**

Our model highlights persistent regional disparities in advanced technology adoption and demonstrates potential for guiding equitable implementation strategies. Its population-based, data-driven approach can be extended to other countries, healthcare settings, and technologies, supporting political decision-making and improved patient access.

**Supplementary Information:**

The online version contains supplementary material available at https://doi.org/10.1007/s11548-026-03723-y.

## Introduction

Technological progress and visceral (gastrointestinal) surgery techniques have consistently enhanced each other’s advancement. This strong interdependence is evident in the rapid progression from open surgical approaches to laparoscopy and, most recently, robotic-assisted procedures within just a century [1] . Emerging technologies such as virtual reality, augmented reality, and artificial intelligence (AI) promise to further improve patient outcomes, safety, and surgical efficiency [2] . In this context, equitable access has been emphasized as a major challenge regarding technological innovation, making universal availability ethically imperative [3, 4].

However, widespread adoption of innovative technologies in clinical practice is often hindered. This is partly related to a poor user acceptance resulting from a lack of trust in AI technologies and concerns about efficiency, ease of use, reliability, and an expected high effort required to integrate them. Consequently, the successful implementation of emerging medical technologies is frequently constrained by high costs, organizational processes, and outdated technical infrastructure, which impede the processing of standardized data for AI systems and autonomous robotics [5, 6].

Furthermore, comprehensive digitalization is a crucial prerequisite for seamlessly integrating emerging technologies into surgical practice. The level of digitalization in healthcare can be objectively assessed using scoring systems such as the Electronic Medical Record Adoption Model (EMRAM), a quantitative tool that rates clinical IT infrastructures and enables comparisons of hospitals or entire countries regarding their digital maturity [7–9]. Thereby, EMRAM mostly focuses on medical record digitization but does not specifically evaluate technological progress impacting gastrointestinal surgery and surgical diagnostics themselves or their actual advancements on patients within the meaning of technological *translation*. The term *translation* thereby refers to the implementation of emerging technologies for the first use in human individuals and ultimately becoming part of clinical routine. Only a few technologies reach the first state and even less actually evolve to a clinical standard [10, 11].

Although the scientific progress of innovative visceral surgical technologies can largely be tracked through publications in literature, quantitatively assessing the real-world adoption and use of these technologies in clinical practice remains challenging [12]. Moreover, in our view, measuring translation means evaluating the number of potential patients who could benefit from a given technology, rather than simply counting hospitals equipped with it or patients already being treated. Meeting this precondition requires a population-based, data-driven modeling of the entire healthcare supply for visceral surgery within a target region of interest [13]. Therefore, we present a novel translation model to assess the technological maturity of visceral surgery in Germany, with potential applicability to other countries.

In this article, we describe the development of our model, present its application to the German population and hospital landscape, and discuss its potential for extension to additional emerging technologies and other countries. Our central research question is: "How are advanced technologies in visceral surgery geographically distributed across Germany, and how can the technological maturity of visceral surgery be objectively assessed and visualized using a translation model to evaluate population access?"

**Table 1:** Data sources and parameter definition

Parameter	Source	Definition
*Surgical procedures*		
Visceral surgery	QR	OPS Codes 5-420 to 5-54 (all)
Endoscopic visceral surgery	QR	OPS Codes 5-420 to 5-54 (endoscopic)
Laparoscopic visceral surgery	QR	OPS Codes 5-420 to 5-54 (laparoscopic)
Robotic surgery	QR	OPS Codes 5-987.[0,1]
*Available devices*		
Angiography	QR	Device code AA01
Computed tomography	QR	Device code AA08
MRT	QR	Device code AA22
PET	QR	Device code AA26
SPECT	QR	Device code AA30
Scintigraphy	QR	Device code AA32
Capsule endsocopy	QR	Device code AA50
Radiofrequence ablation	QR	Device code AA57
3D Laparoscopy	QR	Device code AA72
*Geography and population*		
Population count	Census ’22	Count at latitude/longitude (WGS84)
Shape of German districts	GFAG	Shape-files (WGS84)
Travel-to-treat time	OSRM	Travel times in minutes (isochrones)

*QR: Quality reports of all German hospitals from 2020 and 2023, Census ’22: Census of the German population from 2022, GFAG: German Federal Geodesy Agency, OSRM: Open Source Routing Machine*

## Material and methods

### **Modeling approach**

To build an objective and comparable translation model, we first had to define classes of differing technological maturity and complexity to quantify translation at specific geographic locations. Therefore, we assessed possible class definitions through a survey of 24 technologically experienced surgeons to create technology groups for later model querying. Using the survey results, we grouped available visceral surgery-related technologies and also extrapolated the resulting classes to untranslated and emerging developments. Then we objectively assessed available technologies for clinical routine in every German hospital, for every possible location of population’s residence, using the self-developed modeling framework Geo::Surg and the predefined groupings from the survey. Finally, we applied the model to the German population in 2020 and 2023 to measure the respective degree of technological translation in visceral surgery during this period.

### Survey among visceral surgeons

The survey was conducted among technologically experienced visceral surgeons from the Section for minimally invasive Computer- and Telematics-Assisted Surgery (CTAC) of the German Society of Surgery (DGCH) and from the Technical University of Munich (TUM) Hospital in Munich during November 2025, using the LimeSurvey software, version 3.25.3 [14] . The survey was collaboratively developed by physicians and engineers at the TUM University Hospital in the summer of 2025. It was piloted with three surgeons, and one question was removed after Cronbach’s alpha analysis to enhance internal consistency. The questionnaire and complete methodological details, in accordance with CHERRIES guidelines, are available in the supplementary material [15] . The final survey included ranking questions to evaluate the perceived levels of technological complexity for both currently available and emerging technologies.

Surgical approaches and technologies addressed were *laparoscopy*, *endoscopic surgery*, *angiography*, *computed tomography (CT)*, *magnetic resonance imaging (MRI)*, *capsule endoscopy*, *radiofrequency and thermal ablation*, *robotic-assisted surgery*, *3D laparoscopy*, as well as emerging technologies such as *AI applications* and *autonomous robotics*. Based on the responses, these technologies were grouped into classes according to their perceived level of technological complexity and requirement. The survey also aimed to gather surgeons’ perceptions of emerging technologies and estimate their future potential.

**Fig. 1:**
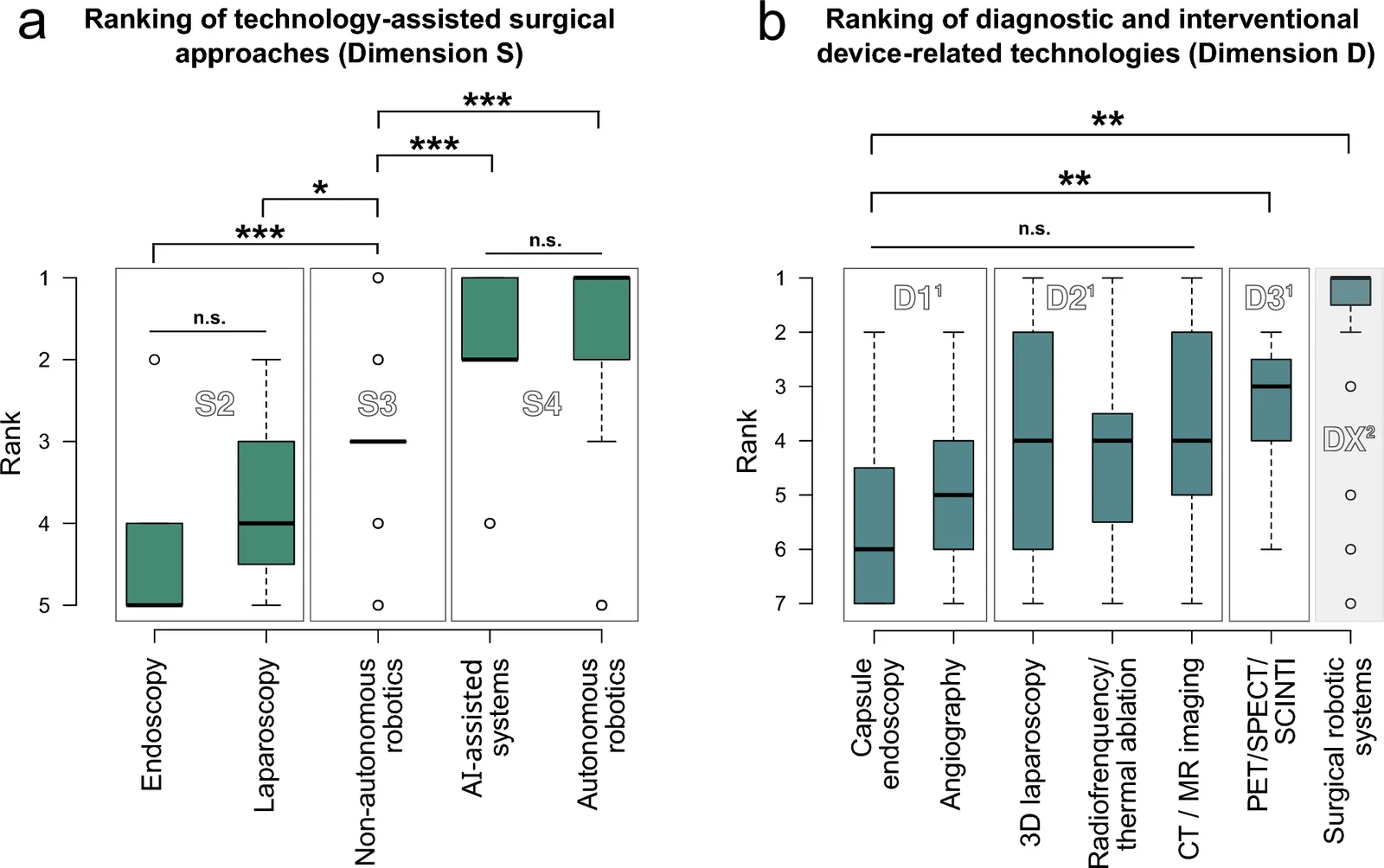
Ranking of surgery-related technologies from the survey among visceral surgeons. (a) Technology-assisted surgical approaches, grouped by ranking (S2–S4); open surgery was defined as S1. (b) Device-dependent technologies, grouped by median ranking (D1–D3).^1^Groups with minor differences were grouped by median. ^2^The application of surgical robots was excluded from dimension D. * p<0.05, ** p<0.01, *** p<0.001, n.s. non-significant

### Development of the translation model

The availability of the technologies of interest and the technology-assisted surgeries performed was objectively derived from the quality reports (n=4266) of all German hospitals from 2020 and 2023. Furthermore, the model drew on data from the 2022 German census and on geographic data from the German Federal Agency for Geodesy (GFAG) and the OpenStreetMap (OSM) project [16–19] . (Table 1) The datasets were integrated in a multimodal translation model using our self-developed healthcare modeling infrastructure Geo::Surg, which was built with the R statistical computing environment, incorporating multiple additional packages for statistical, geospatial, and infrastructural computing [20–23] . This framework is under constant interdisciplinary continuing development within the scope of several projects and is hosted on servers of the TUM University Hospital [24] . It allows for calculating hospital workload and travel times for potential patients, thereby considering infrastructural details such as regional transportation capacities and traffic.

### Application of the model to the German healthcare

Finally, we applied the translation model to the German healthcare provision in visceral surgery as well as to the entire German population. Furthermore, we compared the implementation of technologies in visceral surgery across all 16 German counties. This evaluation also aimed to demonstrate the model’s effectiveness in identifying differences in accessibility and technological maturity across different regions and populations. As profile for measuring accessibility to the technologies of interest, the *by car* option of the OSRM (Open Source Routing Machine) was used, as it represents spatial distances and regional specialties regarding traffic infrastructure the best [25].

### Statistical and comparative assessment

Rather than relating our model for the translation of surgical technologies to former patient collectives or single hospitals, we aimed to apply it to the entire population of observed geographic regions. The primary parameter used to measure the availability and accessibility of a certain technology class was the time an inhabitant of a specific geographic location would have to travel to reach a treatment incorporating the respective technology. All inhabitants with similar access to a specific technology class were grouped within the same *isochrone*, defined as an area sharing comparable travel-to-treat times. This methodology aimed to enable the comparison of different geographic areas and populations. For the statistical assessment of the rankings in the survey among visceral surgeons, the Kruskal-Wallis test was used, with the Wilcoxon rank-sum test as post-hoc analysis and a Bonferroni adjustment for p-values. To assess statistical differences between regions, we used Chi-square statistics. All statistical analyses and model calculations were conducted within the R statistical computing framework, version 4.5.1. The level of significance was set to 0.05.

## Results

### Survey results

24 surgeons completed the survey. 62.5% had more than 5 years of experience in the field of gastrointestinal surgery. Surgical technologies were ranked by perceived technological complexity and requirement (1=highest): 1. Autonomous robotics (Median rank (MR) 1, IQR 1), 2. AI-assisted surgery (MR 2, IQR 1), 3. Non-autonomous robotics (MR 3, IQR 0), 4. Laparoscopy (MR 4, IQR 1.25), 5. Endoscopy (MR 5, IQR 1). Differences between laparoscopy and endoscopy (p = 0.10394) and between autonomous robotics and AI-assisted surgery (p = 1.00000) were not significant. (Fig. 1a)

The equipment of a hospital, specifically the technical devices, was rated by the participants as follows (1=highest): 1. Surgical robotic system (MR 1, IQR 0.25), 2. PET-CT, SPECT, Scintigraphy (MR 3, IQR 1.25), 3. Radiofrequency and thermal ablation technology (MR 4, IQR 1.5), 4. CT/MRI (MR 4, IQR 3), 5. 3D laparoscopy (MR 4, IQR 4), 6. Angiography/DSA (MR 5, IQR 2), 7. Capsule endoscopy (MR 6, IQR 2.25). (Fig. 1b)

According to the participants, the current standard surgical approach for major colorectal surgery requires the highest amount of technological complexity (MR 1, IQR 1), while appendectomy required the least (MR 9, IQR 3).

Most participants assumed that AI could become significant in visceral surgery within 5 years (ME 4, IQR 2), but autonomous surgical robotics was seen as unlikely to do so within that period (ME 2, IQR 1.25). The main barriers to AI and robotics were inadequate infrastructure (79.2%), high costs (70.8%), ambiguous legal aspects (62.5%), lack of training (58.3%), and limited acceptance (50%).

Potential benefits noted as significant included better documentation (70.8%), faster decision-making (66.7%), improved efficiency (66.7%), more precise surgery (54.2%), and fewer complications (54.2%). However, 70.8% of the participants saw little chance for improvement in surgical education/training. Most felt not well-informed about autonomous surgical robotics and AI (ME 2, IQR 1). There was strong consensus that human interaction remains crucial in AI and robotics (ME 5, IQR 1).

### Translation model

Considering the two main sources for surgery-related technologies, namely surgical procedure codes (OPS) and equipment with technological devices reported for each German hospital, we defined two dimensions of the layered technology classification. Dimension *S* incorporated *S*urgical procedures, and dimension *D* considered equipment with visceral surgery-related technological *D*evices.

**Fig. 2:**
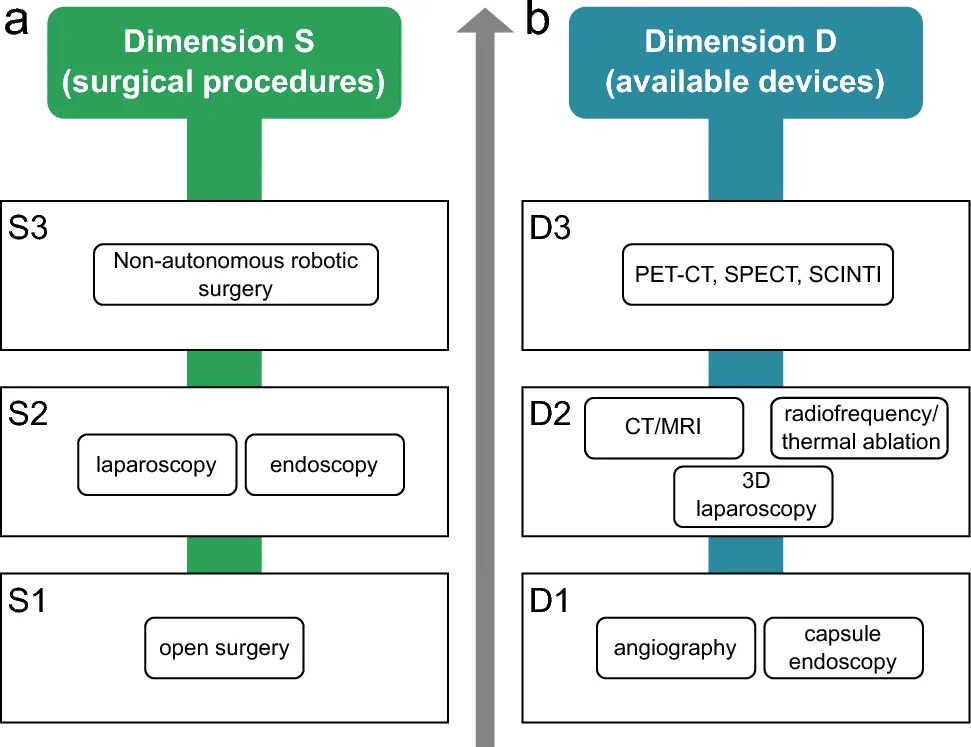
Layered class grouping for visceral surgery technologies. Dimensions S and D are independent; S1 does not correspond directly to D1. Higher technology levels do not require the presence of technologies from lower levels. Unavailable technology groups (S4/D4) have been excluded from the current translation model

For dimension S, we grouped technology-assisted surgical approaches by statistically significant differences in ranking. Open surgery was set as class S1. Based on the survey results, endoscopy and laparoscopy were classified as S2, while currently available non-autonomous robotics were classified as S3. Emerging technologies that are not yet in clinical use were classified as S4 and D4 and excluded from the current model because data to measure actual clinical implementation are not yet available. However, the layered grouping can be readily expanded in future versions once sufficient data becomes available. Final class assignments for dimension S are shown in Fig. 2a.

For dimension D, devices were grouped by median ranking, since most differences were not statistically significant. Capsule endoscopy and angiography formed D1; 3D laparoscopy, radiofrequency/thermal ablation, and CT/MR imaging formed D2; and advanced imaging (PET/SPECT/Scintigraphy) formed D3. Surgical robots were excluded as their use was already represented in class S3 under surgical procedures. (Fig. 2b) Of note, in dimension S and D, higher technology levels do not require the presence of technologies from lower levels.

The layered technology classification was then integrated into the Geo::Surg R framework for querying the final translation model. Thus, the model was capable of simulating how easily each inhabitant can access hospitals offering technology classes S1–S3 and D1–D3, based on travel time from their residence. Therefore, each inhabitant receives a six-number score, one for each technology class, reflecting their travel-to-treat time with lower numeric values indicating greater accessibility. Cumulative regional accessibility is then measured by the proportion of inhabitants assigned to each class and isochrone.

**Fig. 3:**
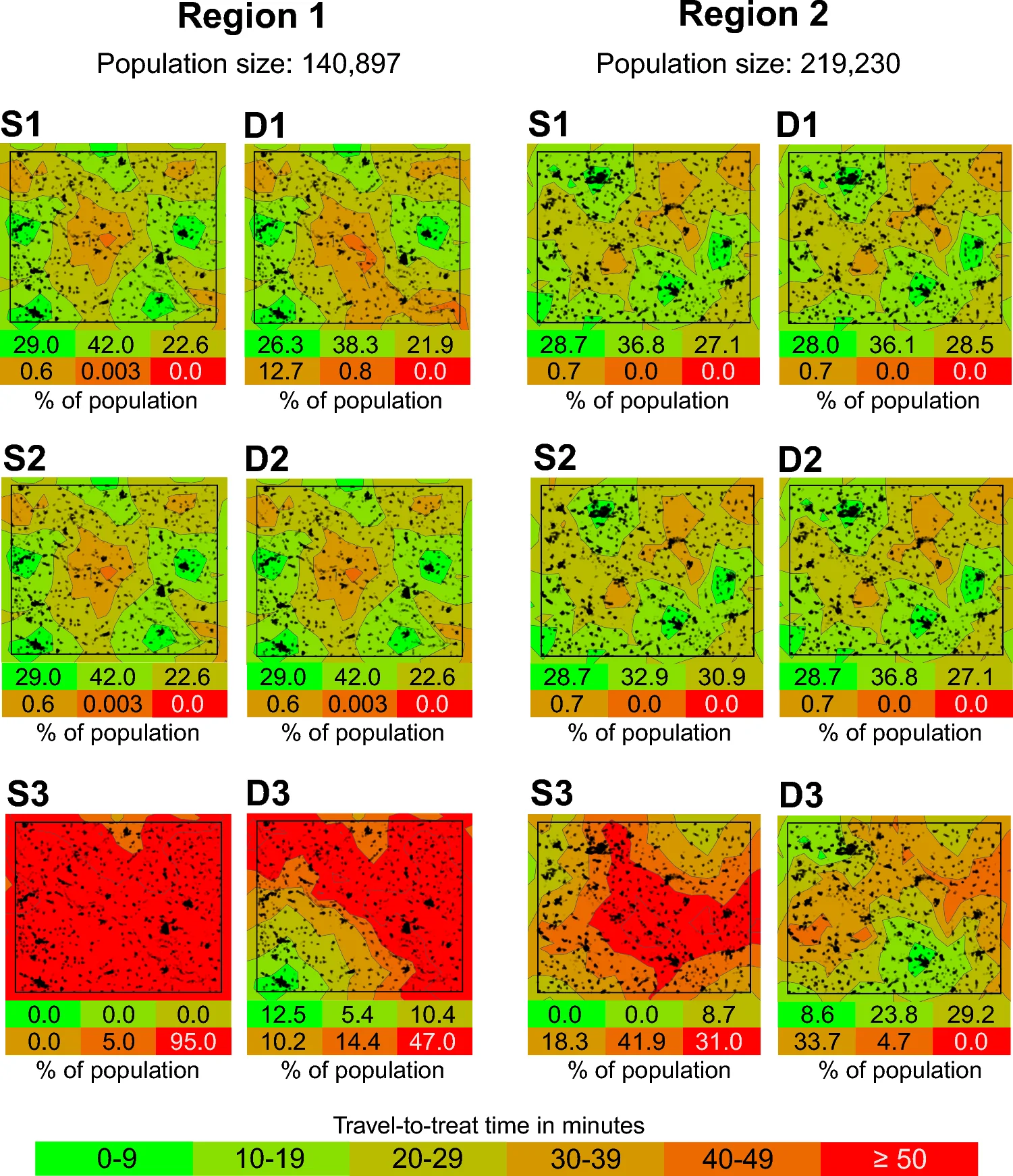
Comparison of technology translation levels between two anonymized randomly selected German regions of the same geographic size in 2023. Black dots show population density; percentages below the maps indicate the share of the population within each isochrone. All differences between the two regions are statistically significant (p<0.001)

### Regional and temporal comparison

**Fig. 4:**
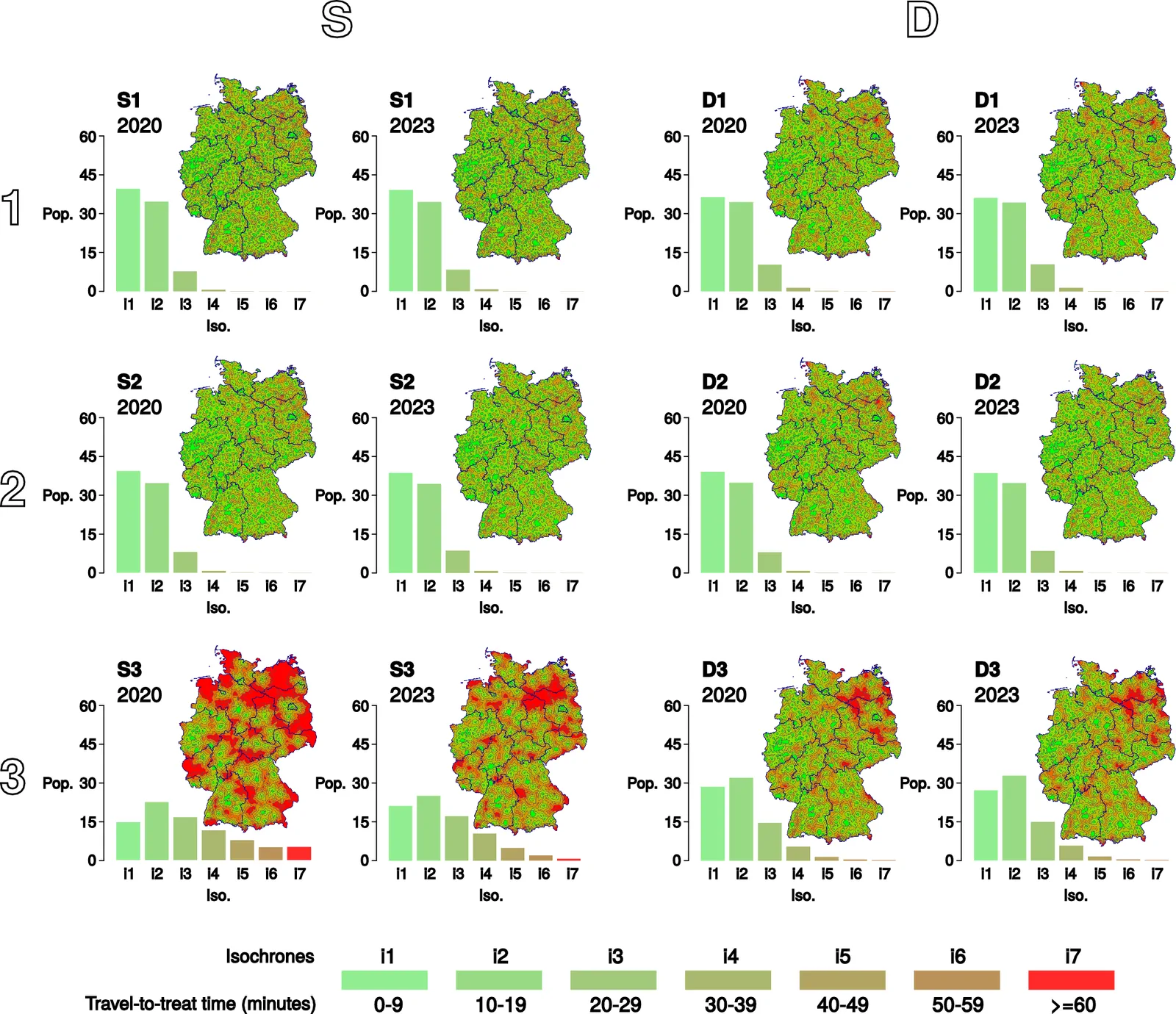
Temporal trends in technology-assisted visceral surgery implementation in Germany (2020 vs. 2023). Pop: population in millions; Iso. (i1–i7): 10-minute isochrones. The changes between 2020 and 2023 regarding the class S3 are statistically significant. (p<0.001)

To assess the translation model’s capability to compare different geographic regions with diverse demographic and infrastructural properties, we defined two anonymized random regions of the same geographic size and shape in Germany and compared the model’s output for both. Region 1 was situated in a rural context, while Region 2 was more urban. (Fig. 3) Compensated for population size (140,897 vs. 219,230) and distribution, the model showed comparable supply for classes S1–S2 and D1–D2, but a significant undersupply for classes S3 and D3 in Region 1 relative to Region 2. Notably, the model also considered the availability of hospitals situated in neighboring regions that exceed the observed regions’ borders. Different population densities are respected by calculating proportions assigned to each class and isochrone rather than absolute numbers. The model can use shapes of any geometry, e.g., bounding boxes or actual administrative borders, to compare the regions within. Additionally, comparing different periods for the same region is also possible, as depicted in the model output for the entire country of Germany, displayed in Fig. 4. Chi-square statistics can be applied using either paired or unpaired methods to assess statistical significance due to the output structure.

### Technology-assisted visceral surgery in Germany

The model included 82.6 million German citizens from the 2022 census, with their places of residence mapped across 3.1 million geographic units. For accessibility modeling, only hospitals with a surgical department (identified by the respective department codes) were selected. In 2020, 1192 hospitals performed visceral surgery, while in 2023, 1141 hospitals conducted surgeries of that kind. In 2020, 1098 hospitals and in 2023, 1047 hospitals encoded laparoscopic procedures. In 2020, 1107 hospitals encoded endoscopic procedures, while in 2023, 1060 hospitals encoded procedures of that kind.

In 2020, 89.87% of the population had to travel less than 20 min to reach any hospital offering visceral surgery in Germany with a stable proportion in 2023 (89.05%). To reach S2 supply, 89.4% in 2020 and 88.5% in 2023 had a traveling time of less than 20 min. The level S3 was reachable in that time frame by 44.8% in 2020 with an increase to 56.8% of the population by 2023. In 2020, 85.7% reached technological equipment of class D1 within 20 min which was stable until 2023 (85.4%) as well as the accessibility to level D2 (89.4% in 2020 and 2023). Level D3 was reachable within 20 min for 73.3% of the German population in 2020 and for 72.5% in 2023. (Model output for each year, class, and isochrone can be found in Fig. 4.)

We also used the model to calculate the technological translation in each of the 16 German counties. The complete statistics for this modeling are shown in Table 2. The highest supply level of surgical technologies was found in *Berlin (BE)*, the capital of Germany, with a proportion of up to 86.7% able to reach classes S1, S2, D1, or D2 within 10 min or less. Classes S3 and D3 were reachable within 10 min by 63.5% and 43.8% of inhabitants, respectively. The lowest supply level regarding technological advancement in visceral surgery was found in the county of *Mecklenburg-Vorpommern (MV)*, with 24.4% of the population at isochrone 1 for S1, S2, and D2, and 12.6%, 23%, and 12.7% for S3, D1, and D3. The statistics indicate a cumulation of advanced technologies in visceral surgery in urban regions compared to a less developed supply in rural regions.

**Table 2:** Technology-assisted visceral surgery across Germany counties in 2023 (Model output)

County code	*SH*						*HH*					
Isochrone	S1	S2	S3	D1	D2	D3	S1	S2	S3	D1	D2	D3
0-9	36.8	35	6.5	29.9	34.5	15.6	74.5	74.4	51.5	65.6	71.4	52.3
10-19	42.9	43.4	33.3	44.5	43.9	44.2	24.7	24.9	45	33.7	27.9	45.6
20-29	18.1	19.3	26.2	20.7	18.5	25.6	0.7	0.7	3.5	0.8	0.7	2.1
30-39	2	2.1	17.4	3.4	2.7	8.6	0	0	0	0	0	0
40-49	0.1	0.1	11.2	0.9	0.3	3.3	0	0	0	0	0	0
50-59	0	0	3.2	0.1	0	1.1	0	0	0	0	0	0
≥ 60	0.2	0.2	2.2	0.5	0.2	1.6	0	0	0	0	0	0
County code	*NW*						*HE*					
Isochrone	S1	S2	S3	D1	D2	D3	S1	S2	S3	D1	D2	D3
0-9	60.6	60.4	33.1	57	60.4	46.2	47.4	47.2	35.7	45.3	47.3	35.1
10-19	36.8	36.8	41.5	38.8	37	42.7	43.5	43.7	29.3	41.5	43.4	43
20-29	2.5	2.7	17	4.1	2.5	8.9	8.5	8.6	19.3	12.5	8.8	15.4
30-39	0.1	0.1	6.2	0.1	0.1	2.1	0.5	0.5	10.8	0.7	0.5	5.4
40-49	0	0	1.8	0	0	0.2	0	0	3.7	0	0	1.1
50-59	0	0	0.4	0	0	0	0	0	1.2	0	0	0
≥ 60	0	0	0.1	0	0	0	0	0	0	0	0	0
County code	*BY*						*SL*					
Isochrone	S1	S2	S3	D1	D2	D3	S1	S2	S3	D1	D2	D3
0-9	42.3	42.1	24.9	39.2	40.7	31.5	56.5	56.5	30	54.9	56.5	43
10-19	44.1	43.6	26.4	44.4	45.3	34.9	36.3	36.3	8.5	30.5	36.3	25.3
20-29	13	13.6	24.4	15.4	13.5	23.7	7.2	7.2	37	13.4	7.2	18.9
30-39	0.5	0.8	15	1	0.6	8	0.1	0.1	20.1	1.1	0.1	12.1
40-49	0	0	6	0	0	1.5	0	0	4.4	0	0	0.7
50-59	0	0	2.6	0	0	0.4	0	0	0	0	0	0
≥ 60	0	0	0.7	0	0	0	0	0	0	0	0	0
County code	*MV*						*SN*					
Isochrone	S1	S2	S3	D1	D2	D3	S1	S2	S3	D1	D2	D3
0-9	24.4	24.4	12.6	23	24.4	12.7	44	43.8	22.4	34.6	44	34.3
10-19	46.8	46.8	22	45.1	46.8	26.6	47.4	47.2	28.1	52.7	47.4	39.9
20-29	22.3	22.3	13.2	23.7	22.3	16.9	8.2	8.5	20.8	10.4	8.2	17.9
30-39	5.4	5.4	20.6	6.6	5.4	18.9	0.3	0.5	14.2	2	0.3	5.9
40-49	0.9	0.9	16.7	1.2	0.9	12	0	0.1	10	0.2	0	1.9
50-59	0.1	0.1	10.1	0.5	0.1	9.2	0	0	4.2	0	0	0.1
≥ 60	0	0	4.8	0	0	3.8	0	0	0.3	0	0	0
County code	*NI*						*HB*					
Isochrone	S1	S2	S3	D1	D2	D3	S1	S2	S3	D1	D2	D3
0-9	41.7	41.4	18	39.3	41.3	30.4	45.1	45.1	18.7	44.3	45.1	39.9
10-19	43.8	43.9	17.3	40.3	43.6	34	53.7	53.7	47.9	54.4	53.7	52.3
20-29	13.2	13.3	24	17.3	13.7	25.3	1.2	1.2	10	1.3	1.2	7.8
30-39	1.1	1.2	21.2	2.8	1.2	8.9	0	0	6.5	0	0	0.1
40-49	0	0	12.5	0.1	0	1.2	0	0	7.4	0	0	0
50-59	0	0	4.5	0	0	0.1	0	0	9.5	0	0	0
≥ 60	0.1	0.2	2.5	0.2	0.2	0.2	0	0	0.1	0	0	0
County code	RP						BW					
Isochrone	S1	S2	S3	D1	D2	D3	S1	S2	S3	D1	D2	D3
0-9	36.3	35.6	15.5	32.3	36.1	24.9	38.3	36.8	22.6	34.4	37.4	27
10-19	48.1	48.2	31.8	42.1	47.4	40.1	47	47.2	30.9	47.4	47.1	42.8
20-29	14	14.6	26.3	22	14.8	22.2	13.6	14.5	24.9	16.2	14.2	21.2
30-39	1.6	1.6	15.9	3.6	1.7	9	1.1	1.5	14.6	1.8	1.2	7.6
40-49	0	0	8.1	0	0	3.4	0.1	0.1	4.9	0.2	0.1	1.4
50-59	0	0	2.1	0	0	0.3	0	0	2.1	0	0	0
≥ 60	0	0	0.4	0	0	0	0	0	0	0	0	0
County code	*BE*						*BB*					
Isochrone	S1	S2	S3	D1	D2	D3	S1	S2	S3	D1	D2	D3
0-9	86.7	86.7	63.5	86.7	86.7	43.8	27.2	27.1	4.1	23.6	27.2	7.8
10-19	13.2	13.2	34.2	13.3	13.2	49.7	50.8	50.8	30.3	48.8	50.8	34.1
20-29	0	0	2.2	0	0	6.2	18.4	18.4	26	19.2	18.4	25.2
30-39	0	0	0	0	0	0.4	3.5	3.5	14.8	6.9	3.5	17.3
40-49	0	0	0	0	0	0	0.1	0.1	10.8	1.4	0.1	9.3
50-59	0	0	0	0	0	0	0	0	8.1	0.1	0	4.9
≥ 60	0	0	0	0	0	0	0	0	5.9	0	0	1.4
County code	*ST*						*TH*					
Isochrone	S1	S2	S3	D1	D2	D3	S1	S2	S3	D1	D2	D3
0-9	39.8	39.8	11	37.9	39.8	20.2	30.4	29.3	10.5	29	30.4	14
10-19	42.3	41.8	26.2	38.3	42.3	29.1	50.5	49.4	21.4	46.3	50.2	37.8
20-29	14.8	15.3	27.1	18.9	14.8	27.8	17.6	19.5	30.1	20.9	17.5	26
30-39	2.9	2.9	17.7	4.7	2.9	15.4	1.5	1.8	25.5	3.8	1.8	16.5
40-49	0.2	0.2	10.1	0.3	0.2	5.9	0	0	8.9	0	0.1	4.9
50-59	0	0	3.7	0	0	1.2	0	0	3.4	0	0	0
≥ 60	0	0	4.2	0	0	0.4	0	0	0.2	0	0	0

*Isochrone ranges are depicted in minutes; Abbreviations for the German counties: SH: Schleswig-Holstein, HH: Hamburg, NW: Nordrhein-Westfalen, HE: Hessen, BY: Bayern, SL: Saarland, MV: Mecklenburg-Vorpommern, SN: Sachsen, NI: Niedersachsen, HB: Bremen, RP: Rheinland-Pfalz, BW: Baden-Württemberg, BE: Berlin, BB: Brandenburg, ST: Sachsen-Anhalt, TH: Thüringen*

## Discussion

In this article, we present a novel modeling approach to objectively measure and monitor the clinical implementation of technology-assisted visceral surgery across geographic and administrative regions. The model is based on the availability and accessibility of these technologies for each citizen within a defined area. We applied this approach to the German population and all German hospitals for 2020 and 2023, providing an objective overview of the dynamics and current state of technological translation in visceral surgery. Technology grouping for querying the final model was informed by a survey of technologically experienced surgeons.

Our literature review found no directly comparable approaches, as most technology-related scoring systems in medicine focus on healthcare digitization and are limited to specific clinical settings [8, 9, 26] . Hence, the model was inspired by established healthcare supply research methods, which use spatial proximity, provider density, or combined ’gravity’ metrics to assess healthcare availability [27] . Traditionally, these geospatial approaches have focused on access to specific treatments or provider workloads for defined population subgroups [13] . We extend these methods to objectively evaluate the translation of surgical technologies across an entire surgical discipline, independent of individual therapies or procedures. This enables our model to potentially serve as an objective tool for measuring the adoption of surgical innovations in routine clinical practice.

We define translation as implementing innovative technologies and ensuring their accessibility for the broadest possible patient population. Our model, therefore, goes beyond the institutional level, measuring availability and accessibility based on each potential patient’s place of residence within a specific geographic region. Among the many ethical considerations, equitable access remains a central principle of medical innovation [3, 4, 28] . By comparing two randomly selected regions in Germany, the final model output demonstrates the ability of our approach to assess areas with distinct characteristics and reveal significant disparities in the adoption of technological advances for visceral surgery. Regional differences between urban and rural areas have been widely discussed in the literature for specific healthcare supply questions [29, 30] . Our model, for the first time, provides an objective, quantitative measure of such disparities in the field of technological translation in visceral surgery.

As expected for a highly developed country, applying the model to Germany demonstrates widespread provision of basic technologies (S1-S2 and D1-D2). Contrarily, robotic surgery (S3) is predominantly available in urban regions, while advanced diagnostics (D3) are also more commonly found in remote areas. For example, national results indicate that *Berlin*, Germany’s most densely populated city-state, offers the highest levels of advanced surgical technologies across all categories, whereas *Mecklenburg-Vorpommern*, the least densely populated and more rural federal state, has the lowest levels [31] . This pattern may reflect implicitly ongoing centralization, with specialized hospitals and advanced technologies concentrated in urban centers [32]. However, rural regions can benefit from proximity to metropolitan areas; for instance, parts of *Niedersachsen* bordering *Hamburg* resulted in much shorter travel times to S3 technologies than other remote areas. Notably, between 2020 and 2023, ongoing implementation of robotic surgery (S3) have led to increased nationwide availability and accessibility. (Fig. 4).

Although our approach did not primarily aim to investigate centralization in surgery, it highlights this phenomenon even in the accessibility of surgery-related technologies. While the ongoing centralization of complex surgical care presents a trade-off between longer travel distances for patients and the benefits of improved clinical outcomes, such as those seen in complex pancreatic surgery, evidence indicates that patients are generally willing to travel further for higher-quality care [33–36]. However our translation model does not specifically address centralization, it may also be well suited to monitor aspects of this tendency, as maintaining a balance between centralization and accessibility is essential, in complex surgery as well as regarding access to surgery-related technologies [37].

As with any modeling approach, our translation model has limitations. First, the grouping classification of technologies for querying the model is based on the assessments of 24 technologically experienced surgeons and may therefore involve some subjectivity. Second, the distribution of emerging technologies, such as AI-assisted surgery or autonomous robotics, cannot yet be demonstrated because the German hospitals’ annual quality reports do not yet contain information about these entities. To support the future application of such models to emerging technologies, we strongly recommend implementing standardized procedure and device codes for AI-supported medicine in clinical practice, along with mandatory reporting of their use.

Notably, inadequate technological infrastructure emerged as the primary barrier to translation, cited by 79.2% of our survey participants. To address this, we believe that integrating combined assessment models is crucial for effective translation monitoring. For example, integrating the EMRAM model with a translation model similar to ours could improve understanding and guidance of implementation processes. Thus, achieving EMRAM level 5 (comprehensive digitized data integration), together with translation classes S1-S3 and D1-D3, may be essential for enabling full AI implementation and autonomous robotics in hospitals in this context [9].

Because our model is compatible with multiple healthcare, demographic, and geographic data, it can be applied to any region or country with similar data availability. This flexibility enables objective cross-country comparisons, as demonstrated by the German counties. Additionally, the model can consider disparity compensation approaches, such as telemedical solutions that virtually bridge geographic distances to connect technologies available at particular hospitals and population groups, thereby further improving their accessibility [38, 39]. To increase the model’s granularity, demographic and epidemiological parameters, such as age structure and disease incidence, could be incorporated, enabling more realistic simulations of patient volumes and, ultimately, more accurate estimates of technology demand among potentially affected patient groups.

In this sense, our model can help to identify local demands and potential barriers to technological translation, thereby supporting health policy decisions to ensure sufficient and equitable patient care.

## Conclusion

There is a notable lack of objective methods to measure the implementation of novel and emerging technologies in visceral (gastrointestinal) surgery. As these technologies advance, ensuring broad and equitable access becomes an ethical imperative. Population-based modeling can serve as a valuable tool for monitoring and guiding translation in this field. Our model, the first of its kind, enables the identification of deprived regions and supports efforts toward providing sustainable and equitable access to technological innovation in healthcare.

## Supplementary Information

Below is the link to the electronic supplementary material.

Supplementary file 1 (docx 44 KB)

## Data Availability

The entire model data set and the results of the survey are available on reasonable request from the authors.

## Code Availability

The entire model data set and the results of the survey are available on reasonable request from the authors.

## References

[1] Berlet M, Fuchtmann J, Jell A, Wilhelm D, Friess H (2025) potential and challenges—robotics and ai in surgery. Gastro-News 12(3):44–53

[2] Rudiman R (2021) Minimally invasive gastrointestinal surgery: from past to the future. Ann Med Surg 71:102922 10.1016/j.amsu.2021.102922 PMC8521242 34703585

[3] Daniels N (2001) Justice, health, and healthcare. Am J Bioeth 1(2):2–16 10.1162/152651601300168834 11951872

[4] Daniels N, Sabin J (2002) Setting limits fairly: can we learn to share medical resources? Oxford University Press, Oxford

[5] Rashid S, Bernhard L, Stabenow S, Spicker E, Haid C, König C, Kramer HL, Pischinger S, Schade D, Fottner J (2025) Bridging the gap between models and reality: development of a research environment for an object-oriented hospital information system to integrate artificial intelligence and robotics into clinical practice. Int J Comput Assist Radiol Surg 20(9):1771–83 10.1007/s11548-025-03470-6 PMC12476447 40608222

[6] Hameed BZ, Naik N, Ibrahim S, Tatkar NS, Shah MJ, Prasad D, Hegde P, Chlosta P, Rai BP, Somani BK (2023) Breaking barriers: unveiling factors influencing the adoption of artificial intelligence by healthcare providers. Big Data Cognit Comput 7(2):105

[7] Li R, Niu Y, Scott SR, Zhou C, Lan L, Liang Z, Li J (2021) Using electronic medical record data for research in a healthcare information and management systems society (HIMSS) analytics electronic medical record adoption model (EMRAM) stage 7 hospital in Beijing: cross-sectional study. JMIR Med Inform 9(8):24405 10.2196/24405 PMC8371484 34342589

[8] Mangiapane M, Bender M (2020) EMR adoption model (EMRAM). Patientenorientierte Digitalisierung Im Krankenhaus: IT-Architekturmanagement Am Behandlungspfad. Springer, Wiesbaden, pp 33–39

[9] HIMSS: Electronic medical record adoption model (EMRAM) | HIMSS. https://www.himss.org/maturity-models/emram/ Accessed 2026-01-05

[10] Marcus HJ, Payne CJ, Hughes-Hallett A, Gras G, Leibrandt K, Nandi D, Yang G-Z (2016) Making the leap: the translation of innovative surgical devices from the laboratory to the operating room. Ann Surg 263(6):1077–1078 10.1097/SLA.0000000000001532 PMC4867108 26727084

[11] Morland C, Pettersen IJ (2018) Translating technological change-implementing technology into a hospital. Int J Product Perform Manag 67(6):1000–1015

[12] Tolikas M, Antoniou A, Ingber DE (2017) The Wyss institute: a new model for medical technology innovation and translation across the academic-industrial interface. Bioeng Trans Med 2(3):247–257 10.1002/btm2.10076 PMC5689495 29313034

[13] Doi S, Ide H, Takeuchi K, Fujita S, Takabayashi K (2017) Estimation and evaluation of future demand and supply of healthcare services based on a patient access area model. Int J Environ Res Public Health 14(11):1367 10.3390/ijerph14111367 PMC5708006 29125585

[14] LimeSurvey Development Team: LimeSurvey—the free and open source survey software tool! LimeSurvey development team, (2025). LimeSurvey Development Team. limesurvey.org

[15] Torrejón-Guirado M-C, San Martín-Erice I, San Martín-Rodríguez L, Lima-Serrano M (2024) Methodological and strategic insights for online survey studies: an analysis based on the cherries checklist. Enfermería Clínica (English Edition) 34(3):207–213 10.1016/j.enfcle.2024.06.001 38852740

[16] Federal Statistical Office of Germany (FSO): [Census 2022]. https://ergebnisse.zensus2022.de/datenbank/online/. Accessed: 2025-08-12

[17] Gemeinsamer Bundesausschuss: quality reports of the German hospitals, years 2020 and 2023. https://www.g-ba.de/themen/qualitaetssicherung/datenerhebung-zur-qualitaetssicherung/datenerhebung-qualitaetsbericht/. Accessed: 2025-10-05

[18] Federal office for cartography and geodesy: shapefiles of Germany and German counties. https://gdz.bkg.bund.de/index.php/default/open-data.html. Accessed: 2025-12-01

[19] OpenStreetMap contributors: planet dump retrieved from https://planet.osm.org . https://www.openstreetmap.org (2025)

[20] R Core Team: R: a language and environment for statistical computing. R foundation for statistical computing, Vienna, Austria (2025). R Foundation for Statistical Computing. https://www.R-project.org/

[21] Giraud T (2022) OSRM: interface between R and the OpenStreetMap-based routing service OSRM. J Open Sour Softw 7(78):4574

[22] Pebesma E (2018) Simple Features for R: standardized support for spatial vector data. R J 10(1):439–446. 10.32614/RJ-2018-009

[23] Wickham H (2016) Ggplot2: elegant graphics for data analysis. Springer. https://ggplot2.tidyverse.org

[24] Berlet M, Jell A, Bernhard L, Wagner L, Reim D, Martignoni M, Demir IE, Feußner H, Friess H, Wilhelm D et al (2024) Geo: surg-a r-framework to optimize regionalization of complex pancreatic surgery based on healthcare, geography and census-based data-a proof of concept. Pancreatology 24:45

[25] Giraud T (2022) OSRM: interface between R and the OpenStreetMap-based routing service OSRM. J Open Sour Softw 7(78), 4574 (2022). 10.21105/joss.04574

[26] Luijten J, Nieuwenhuijzen G, Sosef M, De Hingh I, Rosman C, Ruurda J, Duijvendijk P, Heisterkamp J, Steur W, Laarhoven H et al (2022) Impact of nationwide centralization of oesophageal, gastric, and pancreatic surgery on travel distance and experienced burden in the Netherlands. Eur J Surg Oncol 48(2):348–355 10.1016/j.ejso.2021.07.023 34366174

[27] Stacherl B, Sauzet O (2023) Gravity models for potential spatial healthcare access measurement: a systematic methodological review. Int J Health Geogr 22(1):34 10.1186/s12942-023-00358-z PMC10693160 38041129

[28] Strong VE, Forde KA, MacFadyen BV, Mellinger JD, Crookes PF, Sillin LF, Shadduck PP (2014) Ethical considerations regarding the implementation of new technologies and techniques in surgery. Surg Endosc 28(8):2272–2276 10.1007/s00464-014-3644-1 24962863

[29] Baljepally VS, Metheny W (2022) Rural-urban disparities in baseline health factors and procedure outcomes. J Natl Med Assoc 114(2):227–231 10.1016/j.jnma.2022.01.001 35109969

[30] Herb J, Holmes M, Stitzenberg K (2022) Trends in rural-urban disparities among surgical specialties treating cancer, 2004–2017. J Rural Health 38(4):838–844 10.1111/jrh.12658 35288990

[31] Statistische Ämter des Bundes und der Länder: area and population – population, area and population density. Accessed: 2026-01-02 (2022). https://www.statistikportal.de/de/bevoelkerung/flaeche-und-bevoelkerung

[32] Kugler CM, Gretschel S, Scharfe J, Pfisterer-Heise S, Mantke R, Pieper D (2025) Anticipated effects of centralising complex gastrointestinal surgery in a rural area in Germany-perspective of health professionals: a qualitative study. BMC Health Serv Res 25:824 10.1186/s12913-025-12982-5 PMC12206360 40581641

[33] Finlayson SR, Birkmeyer JD, Tosteson AN, Nease RF Jr (1999) Patient preferences for location of care: implications for regionalization. Med Care 37(2):204–209 10.1097/00005650-199902000-00010 10024124

[34] Nimptsch U, Krautz C, Weber GF, Mansky T, Grützmann R (2016) Nationwide in-hospital mortality following pancreatic surgery in Germany is higher than anticipated. Ann Surg 264(6):1082–1090 10.1097/SLA.0000000000001693 26978570

[35] Krautz C, Nimptsch U, Weber GF, Mansky T, Grützmann R (2018) Effect of hospital volume on in-hospital morbidity and mortality following pancreatic surgery in germany. Ann Surg 267(3):411–417 10.1097/SLA.0000000000002248 28379871

[36] Melnychuk M, Vindrola-Padros C, Aitchison M, Clarke CS, Fulop NJ, Levermore C, Maddineni SB, Moore CM, Mughal MM, Perry C et al (2018) Centralising specialist cancer surgery services in England: survey of factors that matter to patients and carers and health professionals. BMC Cancer 18(1):226 10.1186/s12885-018-4137-8 PMC6389051 29486730

[37] Kelly C, Hulme C, Farragher T, Clarke G (2016) Are differences in travel time or distance to healthcare for adults in global north countries associated with an impact on health outcomes? A systematic review. BMJ Open 6(11):013059 10.1136/bmjopen-2016-013059 PMC5178808 27884848

[38] Berlet M, Fuchtmann J, Krumpholz R, Naceri A, Macari D, Jähne-Schon C, Haddadin S, Friess H, Feussner H, Wilhelm D (2024) Toward telemedical diagnostics—clinical evaluation of a robotic examination system for emergency patients. Digital Health 10:20552076231225084 10.1177/20552076231225084 PMC10777806 38205033

[39] Ferari C, Mitchell K, Crigger C, Zupper S, Wildasin A, Ost M, Hendricks B, Al-Omar O (2022) Bridging the gap—building surgical subspecialty telemedicine clinics in the rural setting. Urology Practice 9(2):126–133 10.1097/UPJ.0000000000000284 37145690

