# Supplementary material for: Technology-assisted visceral surgery: a model-based approach for quantifying translation and accessibility across populations and geographies
Source: Int J Comput Assist Radiol Surg. 2026 Jun 6;21(10):2147–58. doi: 10.1007/s11548-026-03723-y (PMC13723536; doi:10.1007/s11548-026-03723-y)
Supplement: Supplementary file 1 [file 11548_2026_3723_MOESM1_ESM.docx]

# Supplementary

# 1 Translation of technology-based solutions in visceral surgery

Dear participant,

We would like to use the following survey to examine the status of technology-based solutions in visceral surgery in the Federal Republic of Germany. We would like to include your personal opinion on the potential and opportunities of such solutions. If you are a physician working in visceral surgery, we would like to invite you to complete the following online questionnaire and share your assessment of the degree of technological advancement in this subspecialty of surgery. Participation is anonymous and completely voluntary.

If you agree to participate, please check the box indicating your consent to the privacy policy and click “Continue.”

This survey contains 22 questions.

## Demographics

**What age group do you belong to?**

Please select one of the following answers:

- Under 25 years old
- 25–34 years old
- 35–44 years old
- 45–54 years old
- 55–64 years old
- 65 years old or older

Please select one of the following age groups:

**What type of healthcare facility do you currently work in?**

Please select one of the following answers:

- University hospital
- Other tertiary care hospital
- Secondary care hospital
- Primary or general care hospital
- Specialty hospital
- Private hospital
- Other:

Please select the answer that best describes your hospital.

**How large is your hospital or surgical center?**

Please select one of the following answers:

- Less than 200 beds
- 200 to 499 beds
- 500 to 999 beds
- 1,000 or more beds
- Other:

**How long have you been working in surgery?**

Please select one of the following answers:

- Less than 5 years
- 5 to 10 years
- 11 to 20 years
- More than 20 years
- Other:

**What is your current position in your surgical department?**

Please select one of the following answers:

- Resident physician
- Attending physician
- Senior attending physician
- Senior attending physician in a leadership position
- Head of department
- Other:

## Perception of AI and robotics

**To what extent is robotics currently established in your hospital?**

Please select one of the following answers:

- 1
- 2
- 3
- 4
- 5

1 = Not at all established, 5 = Fully established.

**Which surgical procedures are performed robotically/robot-assisted at your hospital?**

Please select all options that apply.

- Reflux and esophageal surgery
- Gastric surgery
- Appendectomy
- Colorectal surgery
- Cholecystectomy
- Other hepatobiliary surgery
- Pancreatic surgery
- Bariatric surgery
- Hernia surgery
- Other:

**To what extent are AI applications currently used in your hospital in the field of visceral surgery?**

Please select one of the following answers:

- Not yet implemented
- Initial pilot projects
- Established in certain areas
- Routinely integrated
- Other:

**In which areas is artificial intelligence currently being used in your hospital?**

Please select all options that apply.

- Preoperative diagnostics (e.g., image analysis, CT/MRI evaluation)
- Surgical planning (e.g., simulation, decision support)
- Intraoperative assistance (e.g., AI-based instrument guidance, detection of anatomical structures)
- Postoperative follow-up
- Prognostic models / outcome prediction
- Speech recognition / documentation / coding
- No use known
- Other:

**How do you assess the potential benefits of artificial intelligence in the clinical routine of your department?**

Please select one of the following answers:

- 1
- 2
- 3
- 4
- 5

1 = No benefit, 5 = Very high benefit.

**Different types of procedures and techniques require different technologies, such as imaging converters, laparoscopic video equipment, or robotic technologies.**

Please rank the following procedure groups in order of their current degree of technologization of their standard procedure. Your personal assessment counts.

- Rank 1 (top): highest degree of technologization of the currently applied methods.
- Rank 10 (bottom): lowest degree of technologization of the currently applied methods.

*NOTE: “Degree of technologization” refers to your personal assessment of the extent and complexity of the technologies required to perform the respective procedure using the standard method currently in practice. For example, the removal of a skin nevus with a scalpel and suture material would be considered a lower degree of technologization than a robotic rectal resection.)*

All your answers must be assigned.

Please rank the boxes in order of preference, starting with 1 and ending with 10.

| ___ | Reflux / esophageal surgery |
| --- | --- |
| ___ | Gastric surgery |
| ___ | Appendectomy |
| ___ | Colorectal surgery |
| ___ | Cholecystectomy |
| ___ | Other hepatobiliary surgery |
| ___ | Pancreatic surgery |
| ___ | Bariatric surgery |
| ___ | Hernia surgery |
| ___ | Endoscopic procedures |

**Please rank the following procedures or systems in order of their required degree of technologization. (Your personal assessment counts.)**

- Rank 1 (top): highest level of technologization.
- Rank 5 (bottom): lowest level of technologization.

(NOTE: “Degree of technologization” refers to your personal assessment of the extent and complexity of the technologies required to perform the respective procedure (currently or in the future).)

All your answers must be assigned.

Please rank the boxes in order of preference, starting with 1 and ending with 5.

| ___ | Autonomous robotics |
| --- | --- |
| ___ | Laparoscopy |
| ___ | AI-assisted systems |
| ___ | Non-autonomous robotics |
| ___ | Endoscopy |

**Please rank the following diagnostic and therapeutic procedures in order of their current degree of technologization.**

- Rank 1: highest level of technologization required.
- Rank 7: lowest level of technologization required.

(NOTE: “Degree of technologization” refers to your personal assessment of the extent and complexity of the technologies required in the standard method currently used. For example, a native X-ray of the wrist would be considered to have a lower degree of technologization than an AI-assisted 3D reconstruction of an MRI.)

All your answers must be assigned.

Please rank the boxes in order of preference, starting with 1 and ending with 7.

| ___ | Angiography system / DSA (digital subtraction angiography) |
| --- | --- |
| ___ | Cross-sectional imaging (CT, MRI) |
| ___ | Capsule endoscopy |
| ___ | Advanced imaging (PET-CT, SPECT, scintigraphy) |
| ___ | Radiofrequency ablation and other thermal ablation procedures |
| ___ | Robot-assisted surgery |
| ___ | 3D laparoscopy |

## Opportunities for the translation of AI and robotics

**How high do you estimate the potential acceptance of AI concepts among your colleagues?**

Please select one of the following answers:

- 1
- 2
- 3
- 4
- 5

1 = Very low, 5 = Very high.

**How likely is it that AI applications will become an integral part of routine surgical practice in the next five years?**

Please select one of the following answers:

- 1
- 2
- 3
- 4
- 5

1 = Very unlikely, 5 = Very unlikely.

**How likely is it that autonomous robotic systems will become an integral part of routine surgical practice in the next five years?**

Please select one of the following answers:

- 1
- 2
- 3
- 4
- 5

1 = Very unlikely, 5 = Very unlikely.

**How would you assess the potential benefits of AI and robotics in visceral surgery over the next five years?**

Please select the appropriate answer for each item:

|  | **1** | **2** | **3** | **4** | **5** |
| --- | --- | --- | --- | --- | --- |
| **AI-based decision support** | ○ | ○ | ○ | ○ | ○ |
| **Robotic surgery** | ○ | ○ | ○ | ○ | ○ |
| **Combination of AI + robotics** | ○ | ○ | ○ | ○ | ○ |

1 = Very low, 5 = Very high.

**What risks or barriers do you see in integrating AI and robotics into routine surgical practice?**

Please select all options that apply.

- High costs / financing
- Lack of evidence
- Acceptance issues
- Inadequate training
- Data protection / liability
- Unclear legal framework
- Technical infrastructure
- Ethical concerns
- Other:

**What advantages do you see in the use of AI and robotics in visceral surgery?**

Please select all options that apply.

- More precise surgery
- Faster decision-making
- Lower complication rates
- Increased efficiency
- Improved documentation
- Enhanced medical training
- No advantages
- Other:

**To what extent do you agree with the following statements?**

Please select the appropriate answer for each item:

|  | **1** | **2** | **3** | **4** | **5** |
| --- | --- | --- | --- | --- | --- |
| **AI can increase surgical precision.** | ○ | ○ | ○ | ○ | ○ |
| **Robotic systems contribute to increased patient safety.** | ○ | ○ | ○ | ○ | ○ |
| **Artificial intelligence will be an indispensable part of surgery in the future.** | ○ | ○ | ○ | ○ | ○ |
| **I feel sufficiently informed about AI in surgery.** | ○ | ○ | ○ | ○ | ○ |
| Despite increasing technologization, the human factor remains crucial. | ○ | ○ | ○ | ○ | ○ |

1 = Strongly disagree, 5 = Strongly agree.

**In your opinion, are there areas where the use of AI or robotics is not appropriate or raises ethical issues?**

Please enter your answer here:

**What specific applications of AI or robotics would you like to see in visceral surgery in the future?**

Please enter your answer here:

Dear participant,

Thank you for participating in our survey.

You may now close your browser window.

*(This survey was originally developed in German and conducted using a digital online form.)*

# 2 CHERRIES checklist for the questionnaire

| ***Checklist item*** | ***Description*** |
| --- | --- |
| *Description of the target population* | *Technologically experienced surgeons with varying levels of experience in visceral surgery from the Department of Surgery at TUM University Hospital, as well as members of the CTAC section of the German Society of Surgery (DGCH), all of whom have access to advanced surgical technologies* |
| *IRB approval* | *IRB approval was not required, as the study involved no patient data or interventional procedures. Only the professional opinions of participants were collected, and there were no ethical concerns.* |
| *Informed consent* | *Informed consent was obtained at the beginning of the online survey. Participants could not access the questionnaire without first reading and agreeing to the data protection section. This section provided information on the survey’s length, the anonymous storage of data, the identity of the primary investigator, and the purpose of the study.* |
| *Data protection* | *The connection to the survey server was SSL-encrypted. The database containing the anonymized data can only be accessed by authorized research team members using two-factor authentication. No identifiers were attached to the records in the database.* |
| *Development and testing* | *The survey was piloted with three visceral surgeons representing different levels of experience. Based on Cronbach’s alpha analysis, one question was removed to improve the reliability of the survey. Electronic functionality was evaluated prior to distributing the invitation link and was repeatedly tested by the research team. The final Cronbach’s alpha for the opinion question section was 0.83, indicating good internal consistency.* |
| *Open survey versus closed survey* | *The survey was conducted as a closed survey, allowing participation only by individuals who received an invitation link.* |
| *Contact mode* | *Potential participants were invited via email using the established mailing lists of the CTAC and the Surgical Department at TUM University Hospital, both of which are routinely used for similar communications.* |
| *Advertising the survey* | *The survey was advertised through the mentioned mailing lists, specifically targeting surgeons involved in visceral surgery. A question about participants' experience in gastrointestinal surgery was included in the demographics section to ensure that only surgeons from this discipline participated. The invitation text is provided in the supplementary materials.* |
| *Web/email* | *The survey was conducted online using the LimeSurvey software.* |
| *Context* | *The LimeSurvey tool used for this survey was hosted on a separate server of the Surgical Department, for this purpose only. There was no website context, and there was no way to find the survey via search engines or other websites.* |
| *Mandatory/Voluntary* | *Participation in the survey was entirely voluntary. Only individuals who actively followed the invitation link were able to access and complete the survey.* |
| *Incentives* | *No incentives were provided to participants for taking part in the survey.* |
| *Time/Date* | *The initial invitation link was distributed on November 18, 2025. The survey remained open for three weeks.* |
| *Randomization of items or questionnaires* | *The items in the sorting questions were presented in a randomized order for each participant to minimize potential order bias.* |
| *Adaptive questioning* | *No adaptive questioning was used; all participants answered the same set of questions.* |
| *Number of Items* | *The survey consisted of 22 questions.* |
| *Number of screens (pages)* | *The questions were distributed over 3 screens.* |
| *Completeness check* | *The LimeSurvey software automatically checked the completeness of each response. Accordingly, survey administrators were able to view the completion status of all submitted questionnaires.* |
| *Review step* | *Participants were able to navigate back to previous questions and review or modify their answers at any time before submitting the survey.* |
| *Unique site visitor* | *Cookies were used by LimeSurvey to ensure that participants could not complete the survey more than once.* |
| *View rate* | *The view rate was not tracked.* |
| *Participation and Completion rate* | *21.4% of the invited surgeons completed the survey.* |
| *Cookies used* | *Cookies were implemented on the first page of the survey and remained active for the entire duration of the survey period, ensuring that each participant could complete the survey only once.* |
| *IP check* | *There was no IP check.* |
| *Log file* | *An automated logfile in LimeSurvey provided an overview of all responses, enabling administrators to monitor survey participation and data collection.* |
| *Registration* | *User registration was not required for survey participation.* |
| *Handling of incomplete questionnaires* | *Only fully completed questionnaires were included in the analysis; incomplete responses were excluded from evaluation to ensure data quality and consistency.* |
| *Questionnaires submitted with an atypical timestamp* | *There were no atypical timestamps.* |
| *Statistical correction* | *No statistical corrections were applied prior to data evaluation. The analysis was performed on raw survey data.* |
